# Nationwide survey on penicillin allergy delabeling among German healthcare professionals: knowledge, attitudes and perceived barriers

**DOI:** 10.1007/s15010-026-02731-z

**Published:** 2026-01-20

**Authors:** Insa Joost, Lukas Tometten, Joanne Barry, Alexandra Weber, Till Koch, Hannah Nuernberg, Anne Moeser, Anette Friedrichs, Barbara Eirmbter, Annette Hennigs, Elham Khatamzas

**Affiliations:** 1 Department of Medical Microbiology and Hospital Hygiene, Duesseldorf University Hospital, Duesseldorf, Germany; 2 Department I of Internal Medicine, Division of Infectious Diseases, Medical Faculty and University Hospital Cologne, University of Cologne, Cologne, Germany; 3 Department of Infectious Disease and Tropical Medicine, Center for Infectious Diseases, University Hospital Heidelberg, Heidelberg, Germany; 4 Antibiotic Stewardship Team and Department of Pharmacy, University Hospital, LMU Munich, Munich, Germany; 5 3rd Medical Clinic for Pneumology, Infectious Diseases and Oncology, Klinikum Itzehoe, Itzehoe, Germany; 6 German Center for Infection Research (DZIF), Partner Site Hamburg-Lübeck-Borstel-Riems, Hamburg, Germany; 7 Hospital Pharmacy, University Hospital Heidelberg, Heidelberg, Germany; 8 Institute of Infectious Diseases and Infection Control, Jena University Hospital, Jena, Germany; 9 Department of Internal Medicine I, Infectious Diseases, University Hospital Schleswig-Holstein, Campus Kiel, Kiel, Germany; 10 Department of Pharmacy, Duesseldorf University Hospital, Duesseldorf, Germany; 11 Division of Infectious Diseases, Albertinen Heart and Vascular Center, Hamburg, Germany; 12 Division of Infectious Diseases, I. Department of Medicine, University Medical Center Hamburg-Eppendorf, Hamburg, Germany; 13 German Center for Infection Research (Deutsches Zentrum Für Infektionsforschung, DZIF), Partner Site Heidelberg University Hospital, Heidelberg, Germany

**Keywords:** Penicillin Allergy Delabeling, Implementation barriers, Antimicrobial stewardship

## Abstract

**Purpose:**

Penicillin allergy (PA) delabeling optimizes antibiotic prescribing and is a recognized antimicrobial stewardship (AMS) tool. We aimed to characterize current practices and knowledge of PA delabeling in German non-allergist healthcare professionals to define barriers and facilitators for a wider implementation of delabeling strategies.

**Methods:**

A nationwide web-based, anonymous survey was distributed to physicians and hospital pharmacists. It covered four domains: demographics, access to AMS, allergy assessment and attitude towards PA delabeling. Responses were analysed using descriptive and inferential statistics.

**Results:**

A total of 504 responses (249 physicians, 255 pharmacists) were analysed. While 86% of respondents were familiar with the concept of delabeling, only 32% had ever performed it, mostly fewer than 6 times in the past year. 12% of physicians regularly took extended allergy histories, and just 2% always used a standardized method. Only ~ 30% of respondents had onsite access to allergy services. Barriers included lack of time, experience and guidance. Pharmacists frequently cited PA management as outside their role. Despite limited experience, 88% of physicians and 71% of pharmacists expressed interest in delabeling if supported by clear algorithms and institutional support.

**Conclusion:**

PA delabeling by non-allergists is of great relevance to physicians and pharmacists in Germany but not routinely integrated in clinical pathways, due to missing guidelines, limited resources and unclear role definitions. However, the results of this study highlight the considerable strong potential for implementing structured delabeling strategies—if they are supported by adequate training, clear protocols and sufficiently resourced antimicrobial stewardship teams.

**Supplementary Information:**

The online version contains supplementary material available at 10.1007/s15010-026-02731-z.

## Introduction:

The rational use of antibiotics is the key element of antimicrobial stewardship (AMS) in the fight against antibiotic resistance [1]. Penicillin and its derivatives are among the most important classes of antibiotics within the framework of rational antibiotic use, as most of them are classified as “Access” Antibiotics by the WHO-AWARE Classification. Penicillin allergy (PA) delabeling is an important AMS tool as 5–19% of hospitalized patients are labeled as penicillin allergic but on evaluation less than 10% of these patients will be truly allergic to penicillin [2, 3]. Use of non-penicillin antibiotics is associated with inferior clinical outcomes, such as increased morbidity and mortality, increased antimicrobial resistance rates and elevated healthcare expenditures [4–7]. Internationally, delabeling strategies range from comprehensive allergy specialist work-up, including serological testing, various skin tests, and graded drug challenges, to risk-adapted approaches based on clinical risk stratification tools such as the PenFAST score [8–11]. In Germany there is no standardized approach to PA delabeling although the German Choosing Wisely Initiative “Klug entscheiden” by the German Society for Internal Medicine (DGIM) recommends to thoroughly evaluate every PA before initiation of antibiotic therapy for instance by using a score [12]. The only available guideline in Germany on diagnostic approach for suspected betalactam hypersensitivity recommends a full allergy workup for every reported PA in an inpatient setting [13]. Given the high number of reported penicillin allergies compared to the limited availability of allergy specialists, a satisfactory evaluation of PAs including the full workup is neither possible in terms of capacity nor economically sensible [14]. Lack of knowledge and awareness form a substantial barrier to implementing new approaches in PA delabeling. Previous surveys have focussed primarily on characterising the knowledge on PA or the practical approach and testing of patients [15, 16]. One survey conducted in a single district general hospital with no specialist allergy service found that the majority of physicians but also pharmacists and nursing staff recognized the need for PA delabeling but emphasized the need for clear recommendations and expressed concerns about the consequences of falsely delabeled patients [17]. We conducted a survey among German healthcare professionals to determine the level of knowledge and management strategies on PA and to identify facilitators and barriers of successful delabeling interventions to aid in wide implementation of delabeling strategies.

## Methods

Interview questions were designed by a team of founding members of the national penicillin allergy network in Germany (PANDA) consisting of 7 hospital-based infectious disease physicians and 3 specialist clinical pharmacists from 7 different university hospitals. The survey comprised four parts: demographics, access to antimicrobial stewardship teams (AMT) and allergy services, practice and knowledge of allergy assessment and PA delabeling (Questionnaire in Suppl. Content). The survey was conducted with SoSci survey (Munich, Germany). The survey was distributed in German. For publication, survey questions were translated into English. An invitation to participate in the anonymised survey and the corresponding link was sent to various healthcare professionals via the email distribution lists of relevant medical and pharmaceutical professional societies. These included the German infection and paediatric infection association (DGI, DGPI), society of hospital pharmacists (ADKA), societies of internal medicine physicians (DGIM, BDI), society of anesthesiology and intensive care (DGAI) and regional networks of antimicrobial stewardship societies (West, Ostwestfalen-Lippe, Rhein Neckar). Links to the survey were also published on the respective society websites as per the discretion of the society’s administration. The survey was open for a duration of 12 weeks (7th November 2023 to 30th January 2024).

A total of 611 persons participated in the survey. After excluding 48 responses that were not involved in direct patient care (did not progress beyond filtering question 4 of survey) and 55 responses that aborted the survey at 30% of survey questions, a total of 504 responses were eligible for further analysis. Questions were binary or multiple choice and included slider controls. Responses were not obligatory; if appropriate multiple answers and free text comments were permitted to capture the likely diversity of participants and practices in Germany, resulting in different total numbers in some sections and therefore to ensure clarity results have been presented both as nearest whole percentage (%) and number (n) of respondents.

### Ethics

The study was approved by the Duesseldorf University Ethics Committee (approval number 2023–2487). The study was conducted in accordance with the principles of the Declaration of Helsinki. Study participants were asked before participation to agree to the privacy policy and further use of their anonymised data after completion of the online survey.

### Statistics

Inferential statistics were used to investigate the populations and their responses to antimicrobial stewardship setting, penicillin allergy knowledge, delabeling practices and perceived barriers. The chi-square test of independence assessed whether the distribution of responses for the physicians and pharmacists are significantly different. Standardized residuals were examined to identify responses significantly contributing to the overall chi-square result. Values exceeding ± 2 were considered notable.

## Results

### Demographics

In total, 504 responses from 249 physicians and 255 pharmacists were analysed. Respondents were from all German Federal States with the three most populous states North-Rhine Westphalia, Bavaria and Baden-Wuerttemberg having the highest representation with 28.2%, 12.3% and 13.7%, respectively (Supplementary Data, Table S1). Among the group of physicians, 57.4% were senior, 29.3% specialist and 13.3% trainee physicians. Specialists from anaesthesiology and intensive care were represented with 29.3% and general medicine with 23.3%. Only 1.6% of respondents were from surgical specialties, 14.4% were internal medicine physicians with 5.2% from infectious diseases. Paediatricians were represented with 7.2%. The working environment was predominantly represented by hospitals with more than 251 beds (83.1%), 18.5% of physicians and 1.6% of pharmacists were working in outpatient settings. 60.4% of respondents were AMT members, with no statistically significant difference between physicians and pharmacists (64.1% physicians vs 57.5% pharmacists) (Table 1).

**Table 1:** Demographics of respondents

			Physicians (%)	(*n*)	Pharmacists (%)	(*n*)
Primary workplace	> 800 beds (university hospital)		24.0	(60)	25.9	(66)
	> 800 beds (non-university hospital)		8.8	(22)	16.9	(43)
	500–799 beds		17.3	(43)	23.5	(60)
	250–499 beds		24.1	(59)	25.9	(66)
	< 250 beds		7.6	(19)	6.3	(16)
	ambulatory		18.5	(46)	1.6	(4)
Level of training	Doctors in training		13.3	(33)		
	Specialist doctor		29.3	(73)		
	Senior doctor		57.4	(143)		
Specialty	General medicine		23.3	(58)		
	Anaesthesiology/intensive care		29.3	(73)		
	Surgical specialty		1.6	(4)		
	Internal medicine (infectious diseases)		5.2	(13)		
	Internal medicine (other)		9.2	(23)		
	Microbiology		3.6	(9)		
	Neurology		5.2	(13)		
	Paediatrics		7.2	(18)		
	Other		15.3	(38)		
AMS team member^1^	yes		64.1	(120)	57.5	(138)
	no		35.9	(67)	42.5	(102)
	not answered		-	(62)	-	(15)

^1^Percentages refer to respondents who answered the question. Missing responses ("not answered") are reported but excluded from percentage calculations

### Access to AMS and allergy services

**Fig. 1:**
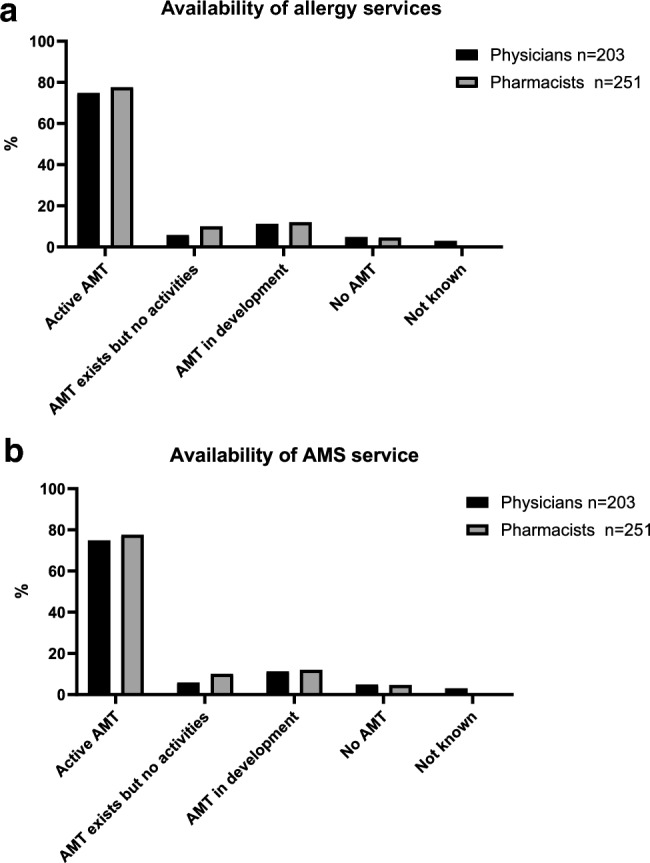
**a** Availability of allergy services. Percentages refer to respondents who answered the question (n). **b** Availability of AMS service. Percentages refer to respondents who answered the question (n). Missing responses ("not answered") were excluded from percentage calculations. *AMT* antimicrobial stewardship team

29.3% and 30.6% of physicians and pharmacists, respectively, had access to allergy services on site, whereas 32.9% of physicians and 16.5% of pharmacists reported the requirement of allergy referrals at another site. 37.8% and 52.9% of physicians and pharmacists, respectively, reported no availability of any allergy specialist services (Fig. 1a). 74.9% of physicians and 77.9% of pharmacists reported an active AMS team; for 17.2% and 22% AMS teams were either not active or still in planning (Fig. 1b).

89.3% of respondents (83.9% physicians, 94.5% pharmacists) had local guidelines for antibiotic treatment of infectious diseases. 88% of respondents stated that their local antibiotic guidelines included alternative antibiotic recommendations for patients with penicillin allergy, however only 31.5% of respondents had access to a local algorithm for the management of patients with penicillin allergy (Table 2).

**Table 2:** Access to local guidelines for management of infections and patients with penicillin allergy

	Yes—Physicians		Yes -Pharmacists		Yes- Total	
	%	*n*	%	*n*	%	*n*
Does your clinic have anti-infective treatment guidelines?	83.9%	(208/248)	94.5%	(241/255)	89.3%	(449/503)
Do these include recommendations for alternative antibiotics for patients with penicillin allergy?	88.1%	(185/210)	88.0%	(212/241)	88.0%	(397/451)
Does your clinic have a guideline for the management of penicillin allergy?	30.9%	(77/249)	32.1%	(82/255)	31.5%	(159/504)

Percentages refer to respondents who answered the question (*n*). Missing responses ("not answered”) were excluded from percentage calculations

### Recording of antibiotic and penicillin allergy

A total of 54.7% of respondents (55.4% physicians, 54.2% pharmacists) indicated that their AMS teams were actively addressing penicillin allergy (data not shown). There were significant differences in the responses between physicians and pharmacists in how allergies were recorded (χ^2^(4, n = 504) = 37.3, p < 0.05) and extended allergy histories were taken (χ^2^(5, n = 504) = 72.0, p < 0.05; Table 3), reflecting distinct understandings, roles and responsibilities in allergy history taking. 12.4% of physicians but only 5.1% of pharmacists reported allergies to only be recorded on specific occasions such as prescription of an antibiotic or perioperatively, on the other hand, 16.5% of pharmacists did not know at all when allergies were recorded (compared to 3.6% of physicians) (Table 3). Significantly more physicians stated that an extended allergy history was always or sometimes taken (11.7% and 33.5% compared to 1.6% and 19.2% of pharmacists, respectively). However, the majority of physicians stated that they rarely or never used a standardized format for acquiring this extended allergy history (Table 3, (χ^2^(5, n = 504) = 81.0, p < 0.05)), whilst, a significant proportion of pharmacists did not know how often or how an extended history was taken (30.2% and 38.4%, respectively).

**Table 3:** Recording of allergy and allergy history taking

	Physicians (%)	(n)	Pharmacists (%)	(n)	%Total	(n)	Statis-tics
Question: “In which patients is the presence of allergies recorded?”							
Routinely	79	(197)	69	(176)	74.6	(376)	
Occasionally	4.8	(12)	5.5	(14)	5.1	(26)	
Only in certain patients, e.g. pre-op	2.4	(6)	2.4	(16)	4.4	(22)	
Only in certain occasions, e.g. before antibiotic therapy	10	(25)	2.7	(7)	6.5	(33)	**
Don't know	3.6	(9)	16.5	(42)	10.1	(51)	**
Question: “How often is an extended allergy history (type of allergic reaction, time, medication, measures taken, etc.) routinely taken?”							
Always	11.6	(29)	1.6	(4)	6.7	(34)	**
Sometimes	33.5	(81)	19.2	(49)	25.8	(130)	**
Seldom	29.7	(74)	28.6	(73)	29.4	(148)	
Never	4.4	(11)	6.7	(17)	5.5	(28)	
Only if antibiotic therapy/Peri-op antibiotic with beta-lactam necessary	16.1	(40)	13.7	(35)	15.1	(76)	
Don’t know	5.6	(14)	30.2	(77)	18.2	(92)	**
Question: “How often is a standardised format used for the extended allergy history (Assessment tool, questionnaire, online tool etc.)?”							
Always	2	(5)	4.3	(11)	3.2	(16)	
Sometimes	13.2	(33)	10.2	(26)	11.7	(59)	
Seldom	21.3	(53)	15.7	(40)	18.6	(94)	
Never	50.2	(125)	23.9	(61)	37.1	(187)	
No extended medical history is taken	5.6	(14)	7.4	(19)	6.7	(34)	
Don’t know	7.6	(19)	38.4	(98)	23.4	(118)	
Question: “Where is an allergy documented?” (multiple answers were possible)							
Medical history paper sheet	50.2	(125)	50.2	(128)	50.6	(255)	
Drug chart (paper)	39.9	(99)	41.9	(107)	41	(207)	
Electronic patient chart	67.5	(168)	70.2	(179)	69.2	(349)	
Allergy alert symbol in the hospital information system	45.4	(113)	45.9	(117)	46	(232)	
Discharge letter	59	(147)	45.5	(116)	52.8	(266)	
Others	4	(10)	4.7	(12)	4.4	(22)	
Question: “Who takes the allergy history?” (multiple answers were possible)							
Medical members of the AMS team	15.3	(38)	13.7	(35)	14.7	(74)	
Other physicians	88.3	(220)	83.5	(213)	86.7	(437)	
Nursing staff	44.2	(110)	58.4	(149)	51.8	(261)	
Pharmacists of the AMS team	2.8	(7)	9.8	(25)	6.3	(32)	
Other pharmacists	3.6	(9)	31.8	(81)	17.8	(90)	
Others	4.8	(12)	3.9	(10)	4.4	(22)	

*AMS* antimicrobial stewardship

69.2% of survey respondents indicated that allergies were recorded in the electronic patient chart, but only 45.6% reported an allergy alert symbol in the electronic hospital information system and only half of respondents document allergies in discharge letters (Table 3). 31.8% of pharmacists stated that peer pharmacists took the allergy history, whereas only 3.6% of physicians made that statement (Table 3).

### Knowledge of penicillin allergy

**Fig. 2:**
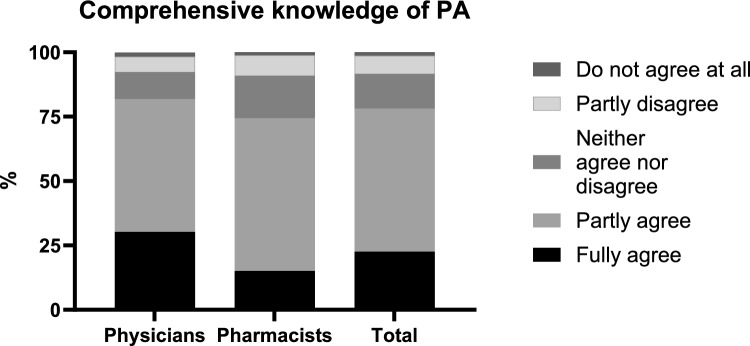
Level of agreement by profession regarding self-assessment of penicillin allergy knowledge (Question: “I have a comprehensive knowledge of penicillin allergy”), *n* = 504

**Fig. 3:**
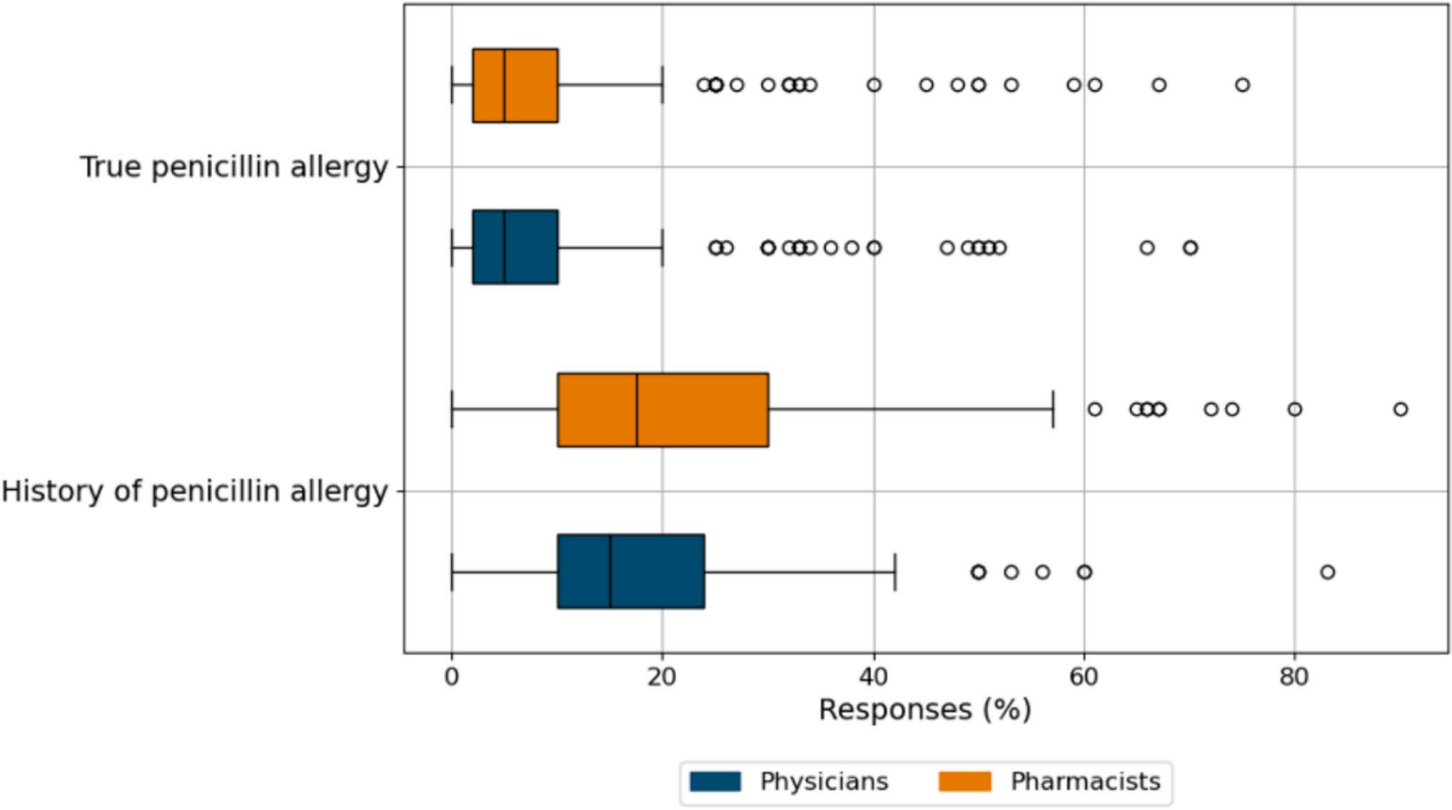
Knowledge of penicillin allergy prevalence among participants. Slider control questions on estimates of patients with a penicillin allergy label and presence of true penicillin allergy

30.1% of physicians, but only 14.9% of pharmacists indicated that they had a comprehensive knowledge of penicillin allergy (standardized residuals = ± 2.6, χ^2^(4, n = 502) = 18.6, p < 0.05) (Fig. 2) but 85% and 86%, respectively, had an understanding of the term penicillin allergy delabeling. However, when asked to estimate the percentage of patients carrying a PA label using slider control questions, the reported estimates were substantially higher (physicians: median 15, IQR 10–24%, pharmacists: median 17.5, IQR 10–30%) than the 6–10% described in the literature [18, 19]. For a true penicillin allergy, the values (median 5%, IQR 2–10% for physicians and pharmacists) were similar to those quoted in the literature [20, 21] (Fig. 3).

### Allergy assessment

**Fig. 4:**
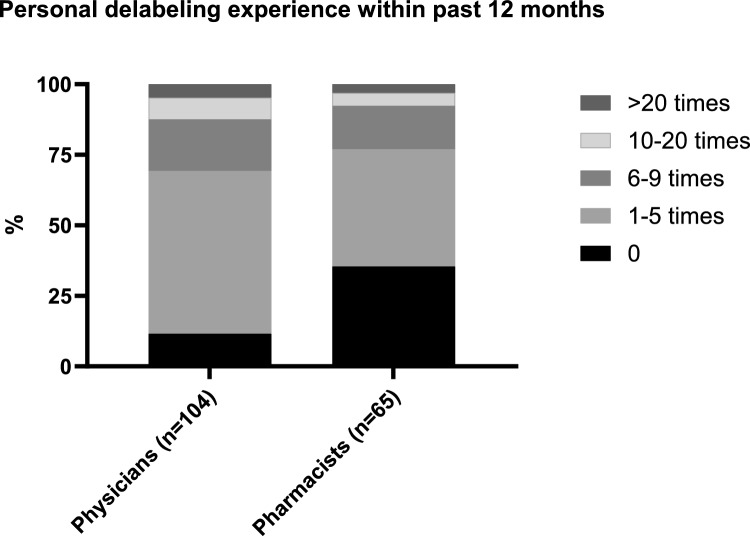
Personal experience of penicillin allergy delabeling among physicians and pharmacists

Although fewer than one third of participants indicated that their institution provided a dedicated guideline for the management of patient with PA (Table 2), 86% of all participants reported being familiar with the concept of 'delabeling', and 41.3% (n = 104) of physicians and 25.9% (n = 65) of pharmacists stated that they had personally performed PA delabeling. Of these, the vast majority (69.2% of physicians and 76.9% of pharmacists) have delabeled no more than five patients in the past 12 months (Fig. 4). Notably, 88.4% of physicians and 71.4% of pharmacists stated that they would perform penicillin allergy delabeling if a clear algorithm and recommendations were available at their institution (χ2(1, n = 481) = 15.7, p < 0.05).

### Barriers

**Fig. 5:**
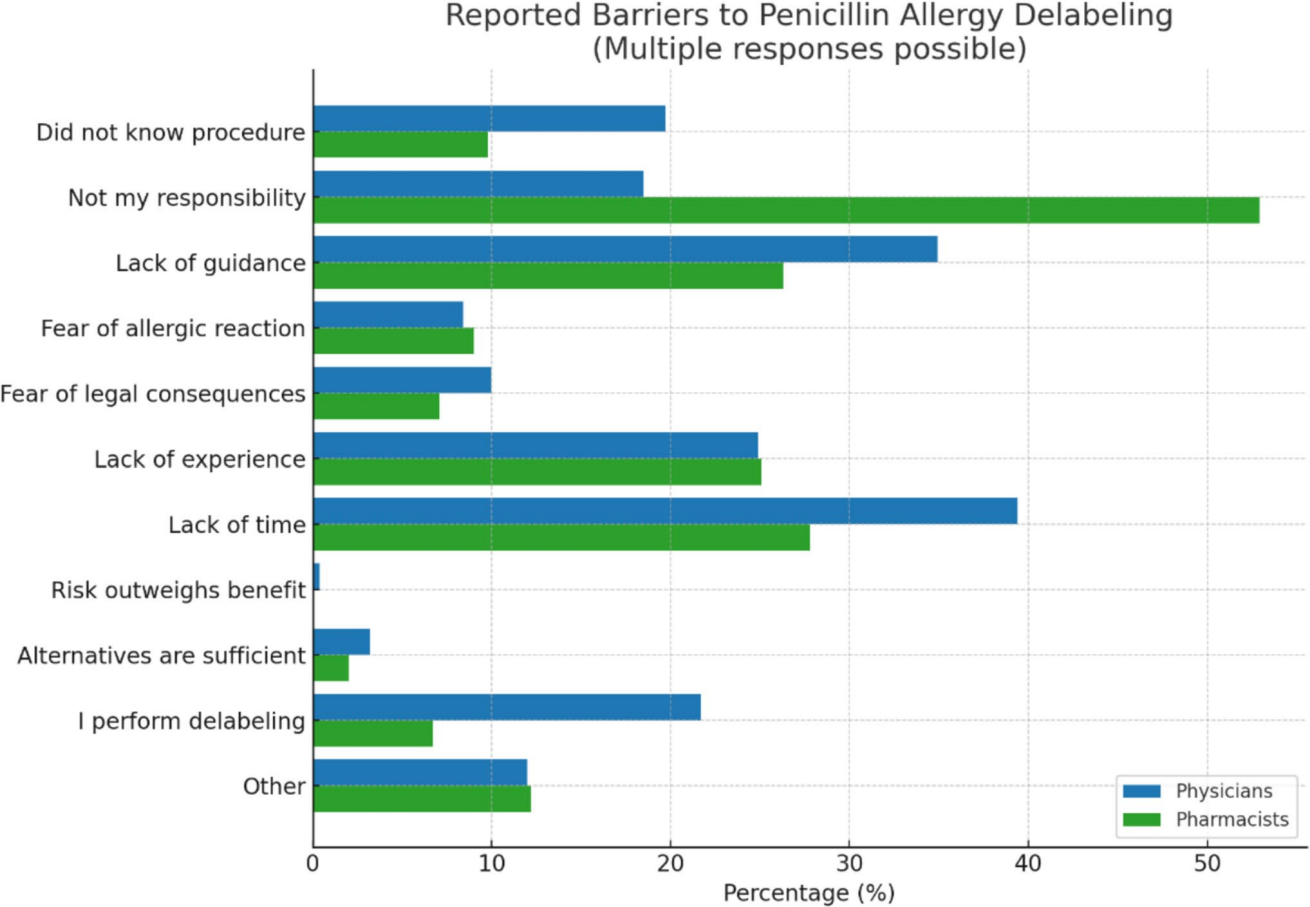
Reasons preventing penicillin allergy delabeling amongst German physicians and pharmacists

When asked for providing reasons that prevented delabeling, a significant proportion of pharmacists stated that it does not fall within their area of responsibility. Lack of time, clear algorithms as well as experience were other reasons for not performing delabeling (Fig. 5). Additional reasons provided in open text boxes included limited access to allergy services, lack of reimbursement, and insufficient institutional support or interest from hospital management (Supplementary Data Figure S1). However, 88% of physicians and 71% of pharmacists stated they would consider delabeling inpatients if provided with a clear algorithm including standardized guidance on history-taking and testing.

## Discussion

This survey aims to give insight into the current situation regarding knowledge, attitude, barriers and facilitators of penicillin allergy delabeling among different groups of German non-allergist health care professionals. A reported penicillin allergy has a significant negative impact on health care outcomes. Implementation of PA delabeling algorithm by multidisciplinary teams is an important quality improvement and antimicrobial stewardship tool and needs to be implemented on a wide scale [22].

Our findings highlight substantial differences in knowledge but also in practices throughout Germany, which may be linked to varying resources and institutional support, emphasizing the urgent need for a national standardized approach. 611 persons answered the questionnaire with 504 being eligible for analysis, showing a considerable interest in the topic.

Currently, no data are available on the extent to which German hospitals are equipped with dedicated AMS teams despite the legal mandate as outlined by the “Infektionsschutzgesetz” (Act on the Prevention and Control of Infectious Diseases in Humans). However, given that > 70% of all respondents reported active AMS services at their workplace and > 50% were active members, it is likely that the survey particularly attracted physicians and pharmacists who were already involved in AMS activities. This self-selection may have introduced a bias toward respondents with a higher level of interest or expertise in the topic.

Despite this potential bias our survey revealed significant gaps in knowledge of penicillin allergy among healthcare professionals. While the majority were familiar with the concept of penicillin allergy delabeling, a significantly smaller number of physicians and of pharmacists considered themselves to have comprehensive knowledge of penicillin allergy management and less than half reported to be actively engaged in this area. Estimates of the prevalence of penicillin allergy were higher than those reported in the literature [8, 9], reflecting a potential overestimation of allergy risk due to lack of knowledge thus contributing to reluctance towards delabeling.

Surprisingly only a minority of all physicians stated that they regularly take an extended allergy history and use a standardized procedure for this assessment both shown to be a prerequisite for any successful delabeling activities [23]. Performing a structured allergy assessment by taking a detailed and thorough allergy history is an important AMS tool itself, enabling the safe application of betalactams even in the absence of established oral provocation protocols [3].

The vast majority of respondents had access to local guidelines covering the treatment of infectious diseases and alternative therapeutic options for penicillin-allergic patients. However, although more than 85% of all respondents were familiar with the term ‘delabeling’, only one third had ever performed delabeling themselves and among these, most had done so less than 6 times within the last 12 months. These findings indicate that the management of penicillin allergy remains largely restricted to the use of alternative therapies, rather than addressing the allergy itself through structured evaluation and delabeling efforts, especially as the majority of respondents were AMS team members and likely more interested and engaged in delabeling activities than potentially the majority of German physicians. Our results suggest that penicillin allergy delabeling is not yet a central focus for providers in Germany, likely due to lack of clear recommendations and inadequate personal resources. Given the broad international recognition of penicillin allergy delabeling as a core AMS responsibility [11, 24], it is crucial that AMS teams nationwide are provided with better support in terms of training and resources.

This interpretation is supported by our findings, which show that the vast majority of participants would proactively engage in delabeling if certain barriers were removed, starting with the availability of a clear algorithm and adequate support at their home institutions, an observation in line with previous surveys [17].

Our survey is the first to not only provide a picture of PA delabeling in the German context but also to include similar numbers of physicians and pharmacists allowing for better insight into the distinct roles each professional group can play in the PA delabeling process. Pharmacists can contribute substantially and support the process by identification of mislabeled patients, thorough history taking, direct delabeling and documentation. Furthermore, they can raise awareness, educate patients and facilitate communication between patients and physicians. Our data demonstrate that this group is highly motivated but requires more support by provision of education and clear algorithms.

Several key barriers to penicillin allergy delabeling were identified in the survey. Among pharmacists, a substantial proportion indicated that delabeling falls outside their area of responsibility, which may be a reflection of the lack of prescribing rights for pharmacists in Germany—in contrast to many other countries, where pharmacists are key providers of penicillin allergy delabeling efforts [24, 25]. This suggests the need for clearer role definitions within multidisciplinary teams. Additionally, both physicians and pharmacists cited barriers such as lack of time, insufficient experience, inadequate institutional support and fear of allergic reactions or legal consequences showing different areas that need to be addressed to allow sustainable changes in PA delabeling practice.

Also, limited access to allergy services across German healthcare institutions might pose an additional barrier. Only approximately one-third of physicians and pharmacists reported access to allergy services on-site, whilst the majority indicated that no allergy specialist services were available on site or at all. This availability is considerably lower than reported in a previous survey [26]. Previous studies have emphasized the importance of integrating allergy testing into antimicrobial stewardship programs to enhance patient safety and optimize antibiotic prescribing [3, 27]. Although the concept of AMS-led penicillin allergy delabeling is intended to be performed without direct involvement of allergology—since only low-risk patients qualify for re-exposure—access to allergology expertise remains valuable for managing complex cases or referrals beyond the low-risk category [22].

This survey has several limitations. First, we could not determine the actual response rate but given the membership numbers of the societies involved this rate is likely low. Second, healthcare providers with interest in PA are more likely to have responded, creating a selection bias. Hence the true knowledge and current practice of penicillin allergy delabeling among non-allergist providers nationwide may differ from that shown by our study, but our experiences as founders of the German Penicillin-Allergy Network (PANDA) concurs with the findings presented. Infectious disease specialists, who could play a pivotal role in PA delabeling, were represented in a very small number within the respondents, which may be due to their limited overall presence in routine care settings or a lower response rate within this group. We did not invite members of allergy and dermatology societies in our survey as we aimed to get a knowledge assessment of non-specialist healthcare providers. Given that PA testing may traditionally be carried out by these specialists in Germany, this may skew the perceived level of knowledge and experience. We selectively invited members of medical specialties traditionally involved in AMS in Germany, therefore experiences and practices of medical professions of other societies such as surgery or neurology, were not addressed. Furthermore, we focused on physicians and pharmacists as health care professionals primarily responsible for medication review and prescribing rights. However, we strongly believe that delabeling is a multidisciplinary effort and further steps in implementing systematically PA delabeling structures must include nursing staff and allied health professionals given their crucial role in identification and communication with patients.

## Conclusion

This nationwide survey provides insights into the current state of penicillin allergy delabeling in Germany in physicians and pharmacists, highlighting several opportunities for improvement. The high level of willingness among healthcare professionals to engage in delabeling activities is encouraging for the broader implementation of structured penicillin allergy delabeling efforts in Germany. However, significant barriers remain, including the current lack of clear recommendations, insufficient financial resources, absence of dedicated financial reimbursement, inadequately staffed AMS teams as well as unclear defined professional responsibilities. Addressing these barriers through the implementation of structured guidelines for penicillin allergy delabeling will strengthen antimicrobial stewardship efforts, ultimately leading to improved antibiotic prescribing, reduced bacterial resistance rates, and better patient outcomes.

## Supplementary Information

Below is the link to the electronic supplementary material.

Supplementary file1 (DOCX 441 KB)

## Data Availability

All data supporting the findings of this study are included in the article and its supplementary material. The underlying raw survey data are not publicly available due to data protection and privacy considerations, but may be made available from the corresponding author upon reasonable request.
