# Supplementary material for: Nationwide survey on penicillin allergy delabeling among German healthcare professionals: knowledge, attitudes and perceived barriers
Source: Infection. 2026 Jan 20;54(2):931–40. doi: 10.1007/s15010-026-02731-z (PMC13021739; doi:10.1007/s15010-026-02731-z)
Supplement: Supplementary file 1 — Supplementary file1 (DOCX 441 KB) [file 15010_2026_2731_MOESM1_ESM.docx]

**Survey on Quality of Care for Penicillin Allergy in Germany**

**1. General Information**

1. **I am aware that this is an anonymous survey. I consent to the use of the data I provide in this survey for the project “Penicillin Allergy Delabeling” and to the publication of the results in anonymized form**.
   a. Yes
   b. No
2. **In which federal state do you work?**
   a. Baden-Württemberg
   b. Bavaria
   c. Berlin
   d. Brandenburg
   e. Bremen
   f. Hamburg
   g. Hessen
   h. Mecklenburg-Western Pomerania
   i. Lower Saxony
   j. North Rhine–Westphalia
   k. Rhineland-Palatinate
   l. Saarland
   m. Saxony
   n. Saxony-Anhalt
   o. Schleswig-Holstein
   p. Thuringia
3. **In what setting do you work?**a. Outpatient
   b. Maximum-care hospital, university, >800 beds
   c. Maximum-care hospital, non-university, >800 beds
   d. Central care, 500–799 beds
   e. Standard care, 251–499 beds
   f. Basic care, <250 beds
   g. Currently not working
4. **Which professional category do you belong to?**a. Physician
   b. Pharmacist
   c. Student
   d. Nursing / Healthcare
   e. Other (free text)
5. **What percentage of your time do you spend in clinical work?**a. Numerical scale

**For physicians:**
**6) Which group do you belong to?**a. Resident
b. Specialist physician
c. Senior physician / head physician

1. **In which specialty do you work?**a. Internal medicine – infectious diseases
   b. Internal medicine – other
   c. Neurology
   d. Pediatrics
   e. Anesthesiology and intensive care
   f. Surgery (incl. ENT, gynecology, urology, ophthalmology, etc.)
   g. General medicine
   h. Microbiology, virology, infection epidemiology
   i. Other
2. **Do you have the possibility to collaborate with a dermatology/allergology department?**a. Yes, on site
   b. Yes, via consultation at another site
   c. No, no direct access to allergology services
3. **Does your hospital have an Antimicrobial Stewardship (AMS) team?**a. Yes, actively working
   b. Yes, but currently not active
   c. Under development
   d. No
   e. Don’t know
4. **If 9) “yes”: Are you a member of the AMS team?**a. Yes
   b. No
5. **If 9) “yes”: Is the AMS team active on the topic of penicillin allergy?**a. Yes
   b. No
   c. Don’t know

**2. Allergy Assessment**

1. **In which patients is the presence of allergies recorded?**a. Routinely in all patients
   b. Occasionally
   c. Only in certain patients (e.g. pre-operative only)
   d. Only on certain occasions (e.g. before antibiotic therapy)
   e. Don’t know
2. **Where is an allergy documented? (multiple answers possible)**a. Medical history paper sheet
   b. Drug chart (paper)
   c. Electronic patient chart
   d. Allergy alert symbol in the hospital information system
   e. Discharge letter
   f. Other: ______
3. **Who takes the allergy history? (multiple answers possible)**a. Medical members of the AMS team
   b. Other physicians
   c. Nursing staff
   d. Pharmacists in the AMS team
   e. Other pharmacists (pharmaceutical history; ward pharmacist)
   f. Other: ______
4. **How often is an extended allergy history (type of reaction, timing, medication, actions taken, etc.) routinely taken?**a. Always
   b. Sometimes
   c. Seldom
   d. Never
   e. Only if antibiotic therapy/Peri-operative antibiotic with beta-lactam necessary
   f. Don’t know
5. **How often is a standardized format used for extended allergy history? (assessment tool, questionnaire, online tool, etc.)**a. Always
   b. Sometimes
   c. Seldom
   d. Never
   e. No extended history is taken
   f. Don’t know
6. **Does your clinic have anti-infective treatment guidelines?**a. Yes
   b. No
   c. Don’t know
7. **Do these include recommendations for alternative antibiotics for patients with penicillin allergy?**a. Yes
   b. No
   c. Don’t know
8. **Does your clinic have a guideline for the management of penicillin allergy?**a. Yes
   b. No
   c. Don’t know
9. **Have you ever witnessed a severe allergic reaction after penicillin intake in a patient?**a. Yes
   b. No

**3. Knowledge About Penicillin Allergy and “Delabeling”**

1. **I have comprehensive knowledge regarding penicillin allergy.**a. Fully agree
   b. Partly agree
   c. Neither agree nor disagree
   d. Partly disagree
   e. Do not agree at all
2. **What percentage of your patients, in your estimation, report a penicillin allergy?**→ Numerical scale
3. **What percentage of these patients, in your estimation, actually have a true penicillin allergy?**→ Numerical scale
4. **Do you have an idea what the term “delabeling” of a penicillin allergy means?**a. Yes
   b. No
5. **Do you personally have experience with penicillin allergy delabeling (structured history, risk stratification, further allergy work-up including oral challenge, and possibly removal of the “penicillin allergy” label)?**a. Yes
   b. No
6. **If 25 “yes”: How often have you performed delabeling in the last 12 months?**a. 0
   b. 1–5
   c. 6–10
   d. 10–20
   e. >20
7. **If 25 “yes”: What diagnostics were performed?**a. Allergy history
   b. Serum IgE measurement
   c. Skin testing
   d. Oral challenge
   e. Other (free text)
8. **Which reasons currently prevent you from performing delabeling? (multiple answers)**a. I did not know the procedure
   b. It is not part of my responsibilities
   c. Lack of clear algorithms/recommendations
   d. Fear of severe allergic reactions
   e. Fear of legal consequences
   f. Lack of experience
   g. Lack of time
   h. In my opinion the benefits of delabeling do not outweigh the risk of a severe allergic reaction during testing
   i. There are, in my opinion, sufficient alternative antibiotics
   j. Other (free text)
9. **Assuming a clear algorithm/recommendation existed, could you imagine personally delabeling patients with penicillin allergy during their inpatient stay (structured history, testing, possible removal of the “penicillin allergy” label)?**a. Yes
   b. No

| *Federal state* | **Physicians %** | **(n)** | **Pharmacists %** | **(n)** |
| --- | --- | --- | --- | --- |
| Baden-Württemberg | 12.0 | (30) | 15.3 | (39) |
| Bayern | 12.9 | (32) | 11.8 | (30) |
| Berlin | 8.0 | (20) | 0.8 | (2) |
| Brandenburg | 1.2 | (3) | 2.7 | (7) |
| Bremen | 1.6 | (4) | 0.0 | (0) |
| Hamburg | 3.2 | (8) | 5.1 | (13) |
| Hessen | 1.6 | (4) | 5.9 | (15) |
| Mecklenburg-Vorpommern | 2.0 | (5) | 2.7 | (7) |
| Niedersachsen | 6.0 | (15) | 12.9 | (33) |
| Nordrhein-Westfalen | 32.5 | (81) | 23.9 | (61) |
| Rheinland-Pfalz | 2.8 | (7) | 3.9 | (10) |
| Saarland | 1.2 | (3) | 2.0 | (5) |
| Sachsen | 8.0 | (20) | 5.1 | (13) |
| Sachsen-Anhalt | 2.0 | (5) | 3.9 | (10) |
| Schleswig-Holstein | 1.6 | (4) | 1.6 | (4) |
| Thüringen | 1.6 | (4) | 1.6 | (4) |
| Not answered | 1.6 | (4) | 0.8 | (2) |
| **Total** | **249** |  | **255** |  |

Table S1. Distribution of respondents


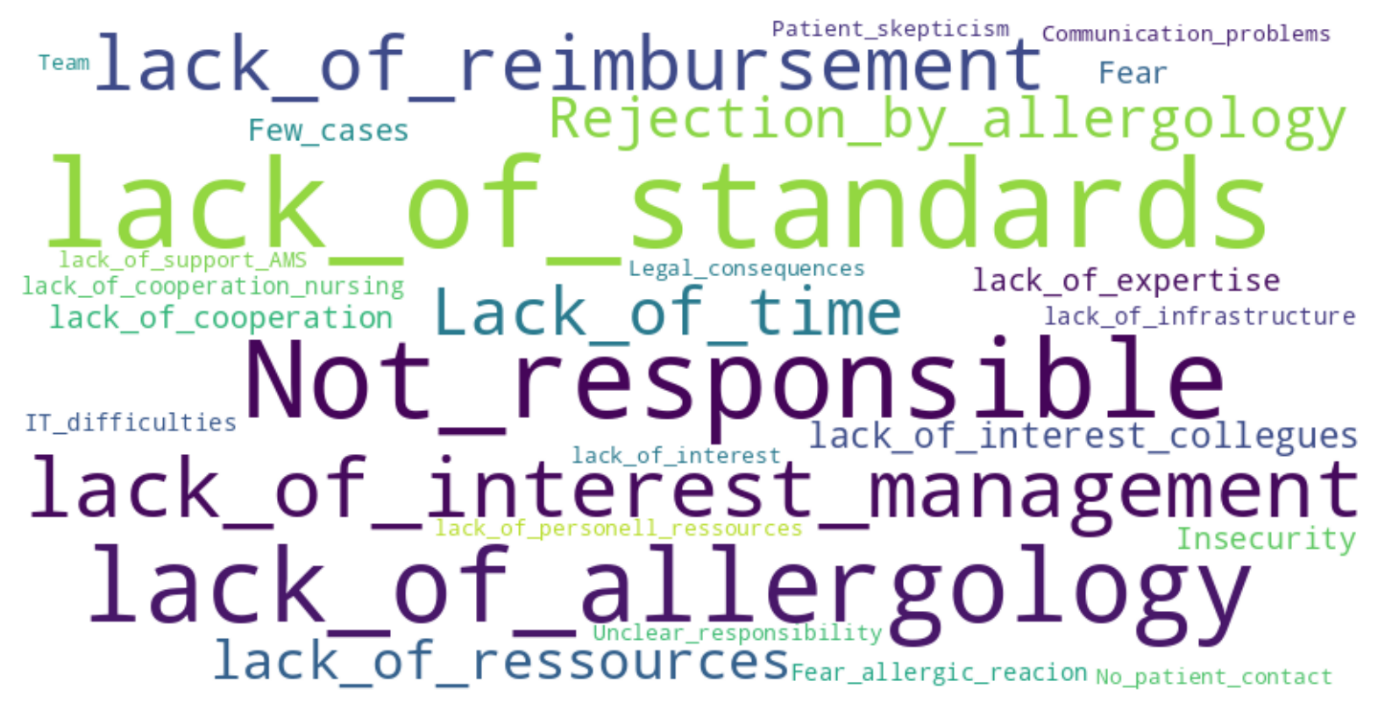


Figure S1. Word cloud based on extracted key words of open text boxes for reported barriers to PA delabeling, visualized with chatgpt.
